# The prognostic importance of CXCR3 chemokine during organizing pneumonia on the risk of chronic lung allograft dysfunction after lung transplantation

**DOI:** 10.1371/journal.pone.0180281

**Published:** 2017-07-07

**Authors:** Michael Y. Shino, S. Samuel Weigt, Ning Li, Vyacheslav Palchevskiy, Ariss Derhovanessian, Rajan Saggar, David M. Sayah, Richard H. Huynh, Aric L. Gregson, Michael C. Fishbein, Abbas Ardehali, David J. Ross, Joseph P. Lynch, Robert M. Elashoff, John A. Belperio

**Affiliations:** 1 Division of Pulmonary and Critical Care Medicine, Department of Medicine, David Geffen School of Medicine at UCLA, Los Angeles, California, United States of America; 2 Department of Biomathematics, University of California at Los Angeles, Los Angeles, California, United States of America; 3 Division of Infectious Diseases, Department of Medicine, David Geffen School of Medicine at UCLA, Los Angeles, California, United States of America; 4 Department of Pathology, David Geffen School of Medicine at UCLA, Los Angeles, California, United States of America; 5 Division of Cardiothoracic Surgery, Department of Surgery, David Geffen School of Medicine at UCLA, Los Angeles, California, United States of America; University of South Carolina School of Medicine, UNITED STATES

## Abstract

**Rationale:**

Since the pathogenesis of chronic lung allograft dysfunction (CLAD) remains poorly defined with no known effective therapies, the identification and study of key events which increase CLAD risk is a critical step towards improving outcomes. We hypothesized that bronchoalveolar lavage fluid (BALF) CXCR3 ligand concentrations would be augmented during organizing pneumonia (OP) and that episodes of OP with marked chemokine elevations would be associated with significantly higher CLAD risk.

**Methods:**

All transbronchial biopsies (TBBX) from patients who received lung transplantation between 2000 to 2010 were reviewed. BALF concentrations of the CXCR3 ligands (CXCL9, CXCL10 and CXCL11) were compared between episodes of OP and “healthy” biopsies using linear mixed-effects models. The association between CXCR3 ligand concentrations during OP and CLAD risk was evaluated using proportional hazards models with time-dependent covariates.

**Results:**

There were 1894 bronchoscopies with TBBX evaluated from 441 lung transplant recipients with 169 (9%) episodes of OP and 907 (49%) non-OP histopathologic injuries. 62 (37%) episodes of OP were observed during routine surveillance bronchoscopy. Eight hundred thirty-eight (44%) TBBXs had no histopathology and were classified as “healthy” biopsies. There were marked elevations in BALF CXCR3 ligand concentrations during OP compared with “healthy” biopsies. In multivariable models adjusted for other injury patterns, OP did not significantly increase the risk of CLAD when BAL CXCR3 chemokine concentrations were not taken into account. However, OP with elevated CXCR3 ligands markedly increased CLAD risk in a dose-response manner. An episode of OP with CXCR3 concentrations greater than the 25^th^, 50^th^ and 75^th^ percentiles had HRs for CLAD of 1.5 (95% CI 1.0–2.3), 1.9 (95% CI 1.2–2.8) and 2.2 (95% CI 1.4–3.4), respectively.

**Conclusions:**

This study identifies OP, a relatively uncommon histopathologic finding after lung transplantation, as a major risk factor for CLAD development when considered in the context of increased allograft expression of interferon-γ inducible ELR- CXC chemokines. We further demonstrate for the first time, the prognostic importance of BALF CXCR3 ligand concentrations during OP on subsequent CLAD risk.

## Introduction

Chronic lung allograft dysfunction (CLAD) is the leading cause of death after the first year post-lung transplantation and the major factor limiting long-term survival.[1] There is increasing evidence that CLAD has two distinct phenotypes: restrictive allograft syndrome (RAS) and bronchiolitis obliterans syndrome (BOS).[2–4] RAS is characterized by restrictive physiology on pulmonary function testing with parenchymal infiltrates, sub-pleural reticulation and septal / pleural thickening on high resolution CT scan (HRCT) of the chest, whereas BOS is characterized by obstructive physiology due to fibro-obliteration of the small airways with air trapping evident on chest HRCT. RAS has been associated with significantly higher mortality compared with BOS.[3,5,6] The pathogenesis of CLAD or its phenotypes, RAS and BOS, remains poorly understood with no effective treatment options. The identification and study of key events which increase CLAD and its phenotypes is a critical first step towards understanding its pathogenesis.

The lung allograft’s response to insults is typically limited to four histopathologic injury patterns that include: acute cellular rejection (AR), lymphocytic bronchiolitis (LB), diffuse alveolar damage (DAD) and organizing pneumonia (OP). Prior studies have established AR [7–18], LB [9,16,19–22] and DAD [23,24] as major risk factors for CLAD development. The association between OP and CLAD or its phenotypes, however, has not been well studied to date. Histopathologically, OP is characterized by excessive proliferation of fibroblasts and granulation tissue predominantly within the alveoli and to a lesser degree, the distal airways. Importantly, OP is similar to the other injury patterns (AR, LB and DAD) in the extravasation and infiltration of mononuclear cells into the area of injury.

CXCL9 (MIG), CXCL10 (IP10), and CXCL11 (ITAC) are interferon-γ inducible ELR- CXC chemokines (CXCR3 ligands) which signal through a shared G protein-coupled receptor, CXCR3. These chemokines are induced by interferon-γ, act as potent chemoattractants for mononuclear cells (e.g., activated T-cells and NK cells) and are major mediators of the Type I immune response. Our group has previously demonstrated the importance of CXCR3/ligand biology in the pathogenesis of AR [25] and CLAD [26] in both animal and human studies.

In the current study, we hypothesized that BALF CXCR3 ligand concentrations during OP would have prognostic value in predicting the risk of subsequent CLAD development. Specifically, we hypothesized that CXCR3 ligand concentrations would be elevated during OP and that episodes with higher BALF CXCR3 ligand concentrations would be associated with higher CLAD risk. Given the alveolar and intra-parenchymal localization of the injury, we furthermore hypothesized that OP would be predictive of the restrictive phenotype of CLAD, RAS.

## Materials and methods

With IRB approval, we performed a retrospective review of all lung transplant recipients (LTRs) at UCLA between January 1, 2000 and December 31, 2010. Participants provided full written consent for their medical records to be reviewed for this study. LTRs received a surveillance bronchoscopy with bronchoalveolar lavage (BALF) and transbronchial biopsy (TBBX) at 1, 3, 6 and 12 months post-transplant, as well as during episodes of clinical deterioration. One of three pulmonary pathologists interpreted the biopsies according to the International Multidisciplinary Consensus Statement on Idiopathic Interstitial Pneumonias (OP and DAD)[27], and the International Society for Heart and Lung Transplantation criteria (AR and LB)[28,29] Biopsy data were coded for the presence or absence of OP, DAD, LB and AR (grade A1 or greater). TBBXs with no histopathologic evidence of allograft injury were considered “healthy”. Ungradable biopsies were considered to be a missing value. Study participants were aged 20 to 79 with a mean age of 60.

Immunosuppression, anti-microbial prophylaxis and treatment of acute rejection were administered in accordance with UCLA protocol as previously described.[30] Treatment for OP was by discretion of the transplant pulmonologist and included: methylprednisolone, IVIG, plasmapheresis, basiliximab, ATG or no treatment. Serial spirometry was performed at least quarterly. CLAD was defined as a sustained 20% drop in the forced expiratory volume in 1 second (FEV1) from the average of the two best post-transplant FEV1 measurements.[8,31] In a subset analysis of double LTRs, CLAD was further categorized as RAS or BOS based on Sato and colleagues’ 2013 definition utilizing spirometry [32] and chest computed tomography (CT). RAS was defined as ΔFVC%/ ΔFEV1% > 0.5 and chest CT showing ground glass opacification, interstitial reticulation or interlobular septal thickening. Recipients with CLAD who did not fulfill RAS criteria were considered to have the BOS phenotype. Those who did not have a chest CT within 3 months of CLAD diagnosis were excluded from this subset analysis.

LTRs consented to the collection of BALF fluid for research purposes at the time of their bronchoscopies. Three 60 ml aliquots of isotonic saline were instilled into the sub-segmental bronchus in the lingula, right middle lobe or area of interest and pooled. After centrifugation, the supernatant was collected and stored unconcentrated at -80°C. CXCR3 ligand concentrations were measured using CXCL9, CXCL10 and CXCL11 bead assays (Millipore, Billerica MA). CXCR3 ligand concentrations were compared between OP and “healthy” biopsies using linear mixed effects models to account for repeated measurements from the same individuals.

To evaluate the effect of OP on CLAD risk, univariable proportional hazards models for time to CLAD were constructed with cumulative time-dependent counts for OP. The multivariable model was adjusted for the other known histopathologic predictors of allograft injury (DAD, AR and LB) using cumulative time-dependent counts. To explore the impact of BALF CXCR3 ligand elevation during OP on subsequent CLAD risk, a time-dependent cumulative variable for OP was created using quartiles of CXCL9, CXCL10 and CXCL11 concentrations. For example, using the first quartile cutoff, “high-CXCL9 OP” would increase from 0 to 1 during the first episode of OP with BALF CXCL9 concentration greater than the 25^th^ percentile. At the second episode “high-CXCL9 OP”, the variable would increase from 1 to 2. Analyses were performed with SAS (v9.4).

## Results

### Histopathologic findings

**Fig 1:**
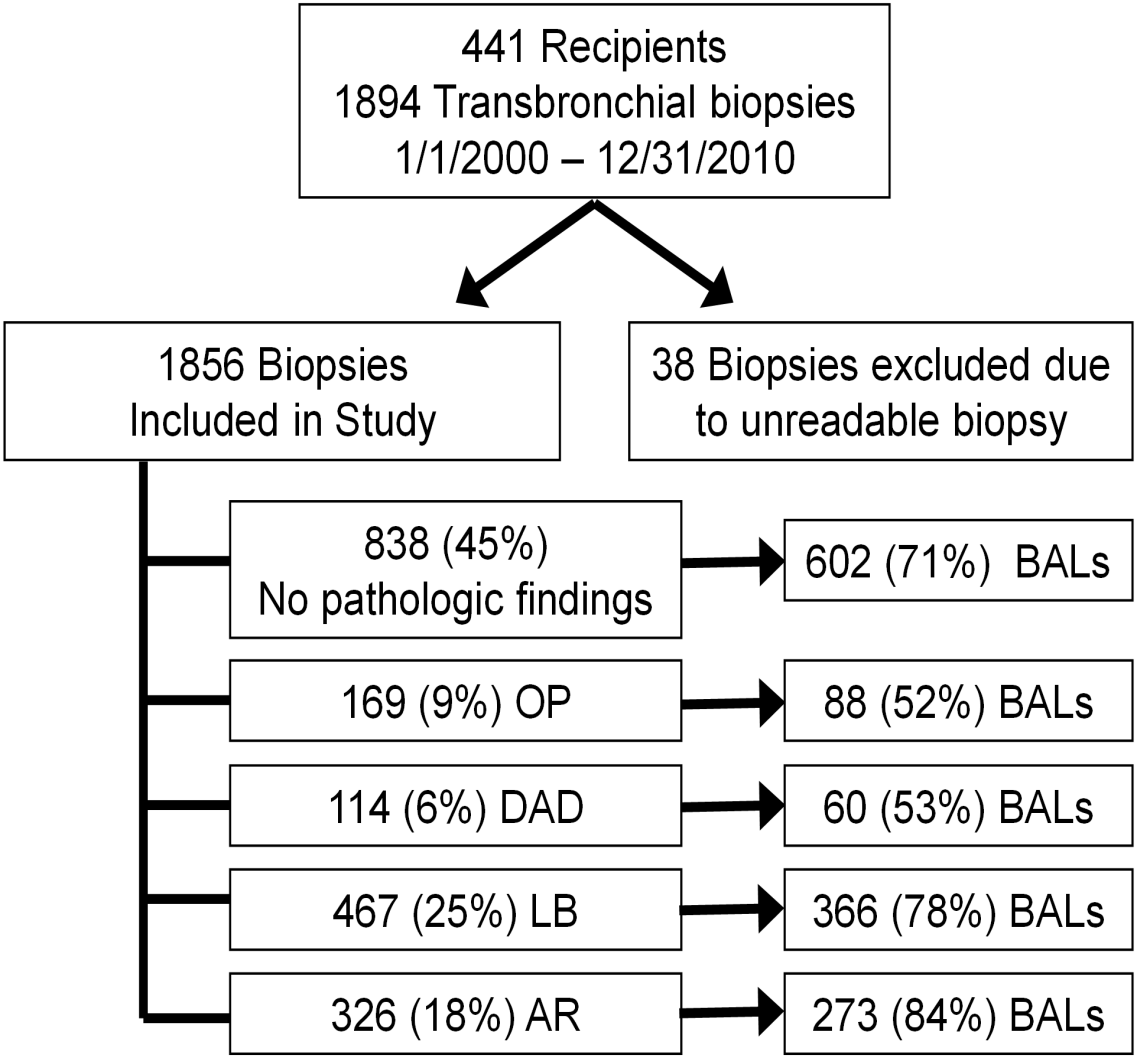
Study profile. OP = organizing pneumonia; DAD = diffuse alveolar damage; LB = lymphocytic bronchiolitis; AR = Acute rejection (Grade A1 or higher); BALs = bronchoalveolar lavage.

There were 1894 bronchoscopies with TBBXs from 441 LTRs evaluated. There were 169 (9%) biopsies with OP, 114 (6%) with DAD, 565 (30%) with LB and 391 (21%) with AR (Fig 1). Three hundred and three (16%) TBBXs had concurrent injury patterns. LB and AR occurred together most frequently (n = 193, 10%). OP occurred with LB (n = 79, 4%), AR (n = 45, 2%) and DAD (n = 30, 2%). Eight hundred thirty-eight (44%) TBBXs had no histopathology and were classified as “healthy” biopsies. Table 1 shows demographic and clinical characteristics of recipients by whether they ever developed OP. Most variables were evenly distributed between those who developed OP vs those who did not.

**Table 1:** Baseline patient characteristics. By never / ever developed organizing pneumonia.

	Never OP		Ever OP		p-value
	n (%)	%	n (%)	%	
Number of patients with:	324	73%	117	27%	
Median age	58		56		0.06
Male gender	191	59%	68	58%	0.88
Single lung transplant	145	45%	44	38%	0.18
Diagnosis					
Restrictive ILD	183	57%	67	57%	0.88
COPD / AAT	103	32%	30	26%	0.21
CF / bronchiectasis	21	6%	6	5%	0.60
Other	17	5%	14	12%	0.01
Induction					
ATG	182	56%	65	55%	0.91
Basiliximab	141	43%	51	44%	0.99
None	1	1%	1	1%	

Definition of abbreviations: OP = organizing pneumonia; ILD = interstitial lung disease; COPD = chronic obstructive pulmonary disease; AAT = alpha-1 antitrypsin deficiency; CF = cystic fibrosis; ATG = thymoglobulin.

### Etiology of organizing pneumonia

We explored the etiology of OP by reviewing microbiology cultures, TBBX stains and cultures for organisms, radiographic studies and clinical notes from the treating physician. In the majority of cases (n = 117, 69%) there was no discernable etiology for the OP. When the etiology for OP could be identified, the most common was bacterial infection (n = 25, 15%). Bacterial etiologies included: Pseudomonas aeruginosa, Stenotrophomonas maltophilia, Methicillin-sensitive Staph aureus, Enterobacter cloacae, Acinetobacter baumannii, Escherichia coli, Nocardia, Serratia marcescens and Vancomycin-resistant Enterococcus. There were 9 (5%) viral causes of OP including: parainfluenza, CMV, human metapneumovirus, corona/echovirus and coxsackie/echovirus. Other etiologies included fungus (n = 4, 2%), Aspergillus fumigatus, Aspergillus niger, Coccidioidomycosis and Scedosporium prolificans) and non-tuberculous mycobacterium (n = 5, 3%), Mycobacterium abcessus and Mycobacterium avium complex). Nine (5%) episodes of OP were associated with high-grade (grade 3) acute rejection.

### BALF CXCR3 chemokine elevations during organizing pneumonia

We hypothesized that BALF CXCR3 ligands would be elevated during episodes of OP. To test this hypothesis, we assayed 690 BALF samples from 324 recipients. There were 602 “healthy” samples from 300 recipients and 88 OP samples from 72 recipients. The median BALF CXCL9, CXCL10 and CXCL11 concentrations were significantly higher during OP compared with “healthy” biopsies (Table 2). CXCL9, CXCL10 and CXCL11 concentrations for OP and “healthy” biopsies were 1140 vs 335 (p = 0.0015), 287 vs 135 (p = 0.0005), and 67 vs 62 pg/ml (p = 0.0142), respectively. Given the high correlation among the three chemokines, a principal component analysis was performed to assess the three chemokines in aggregate. The first principal component (PC) was estimated as: *PC = 0*.*49114 × log*_*10*_*(CXCL9) + 0*.*52091 × log*_*10*_*(CXCL10) + 0*.*27251 × log*_*10*_*(CXCL11)*. The PC accounted for 57% of the total chemokine variation. The PC also demonstrated significant chemokine elevations during OP compared with “healthy” biopsies: -0.049 vs -0.123 (p = 0.0001), respectively.

**Table 2:** Median BAL CXCR3 ligand concentrations. By healthy biopsies vs. organizing pneumonia.

	Healthy pg/ml	OP pg/ml	p-value ƚ
**CXCL9**	335	1,140	0.002
**CXCL10**	135	287	0.001
**CXCL11**	62	67	0.014
**PC** ƚƚ	(0.123)	(0.049)	0.001

Definition of abbreviations: BAL = bronchoalveolar lavage; pg/ml = picogram/milliliter.

^ƚ^ Mixed effects model comparing OP vs healthy biopsies.

^ƚƚ^ PC: First principal component of CXCL9, CXCL10 and CXCL11.

PC = 0.357×log(CXCL9)+0.439×log(CXCL10)+0.386×log(CXCL11).

### Cellular sources of the chemokines and their shared receptor CXCR3

**Fig 2:**
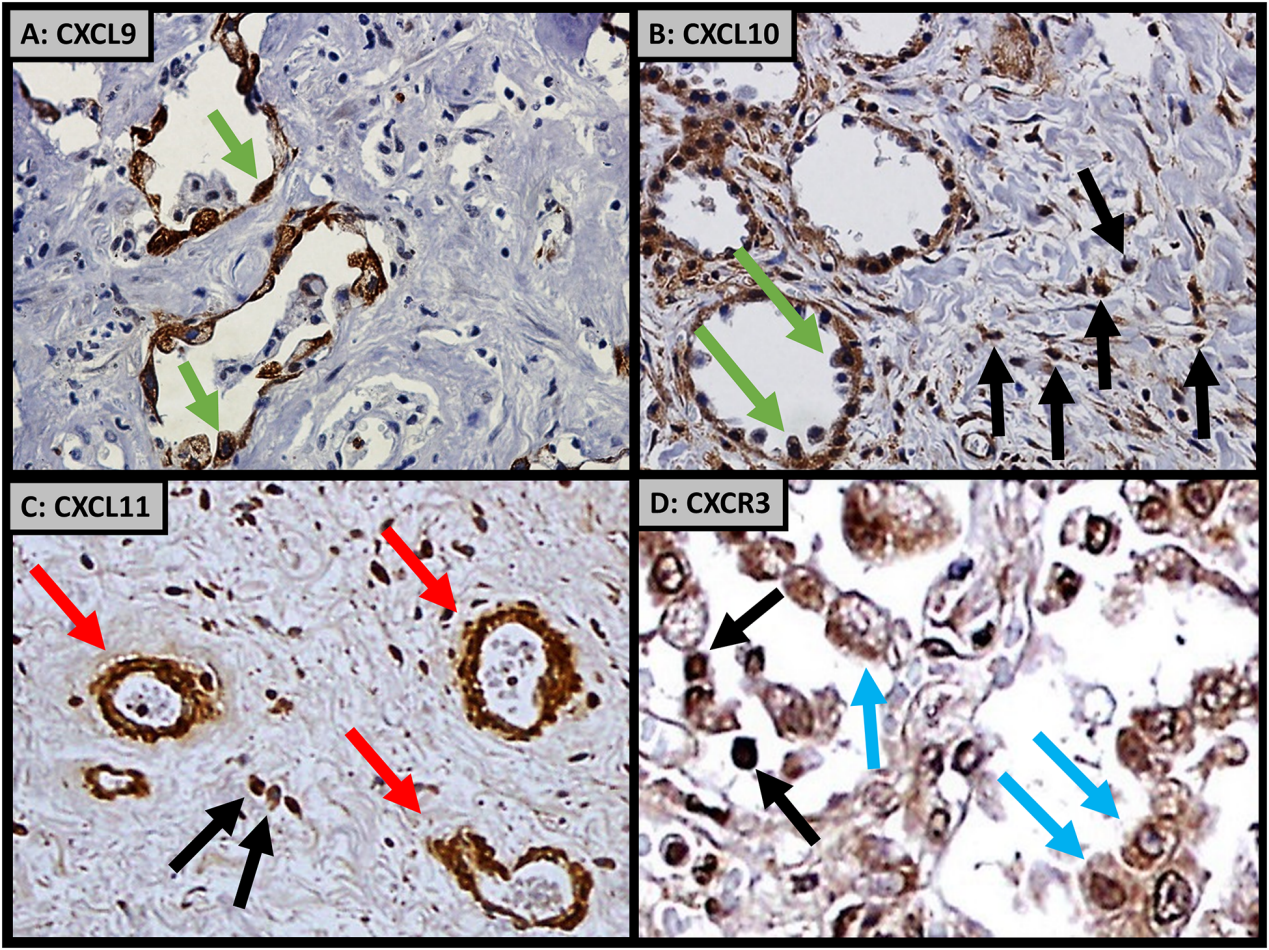
Immunohistochemistry demonstrating A) CXCL9 and B) CXCL10 expressed from allograft alveolar pneumocytes (green arrows) and interstitial infiltrating mononuclear cells (black arrows). C) CXCL11 expressed from allograft pulmonary vascular endothelial cells (red arrows). D) CXCR3 expressed from allograft infiltrating mononuclear cells (black arrows) and macrophages (blue arrows).

We used IHC on lung biopsy tissue to determine the cellular sources of these chemokines and their shared receptor, CXCR3, during OP. Both CXCL9 and CXCL10 were expressed predominantly by reactive type 2 pneumocytes and infiltrating mononuclear cells (Fig 2). In contrast, CXCL11 was primarily expressed by vascular endothelial cells and infiltrating mononuclear cells. CXCR3 was predominantly expressed by allograft infiltrating mononuclear cells and macrophages.

### Risk of CLAD after organizing pneumonia

To evaluate the impact of OP on CLAD risk, univariable and multivariable proportional hazards models were constructed with time-dependent cumulative counts for OP as well as the other histopathologic injury patterns AR, LB and DAD. OP predicted CLAD development in the univariable model (HR 1.5 95% CI 1.1–2.1), but lost significance after multivariable adjustment for AR, LB and DAD (Table 3).

**Table 3:** Cox proportional hazards model for CLAD. Using BAL CXCL9 concentrations cutoffs.

	Univariable		Multivariable ƚ	
	HR (95% CI)	p-value	HR (95% CI)	p-value
**OP**	1.55 (1.13–2.12)	0.007	1.34 (0.97–1.86)	0.077
**OP with CXCL9 > 25th%**	1.64 (1.11–2.41)	0.013	1.38 (0.92–2.06)	0.119
**OP with CXCL9 > 50th%**	2.19 (1.42–3.35)	0.001	1.90 (1.22–2.94)	0.010
**OP with CXCL9 > 75th%**	2.32 (1.37–3.92)	0.002	1.83 (1.06–3.16)	0.031

Definition of abbreviations: CLAD = chronic lung allograft dysfunction; HR = hazards ratio;CI = confidence interval; OP = organizing pneumonia; % = percentile.

^ƚ^ Multivariable model adjusted for diffuse alveolar damage, acute rejection, and lymphocytic bronchiolitis.

### Impact of high BALF CXCR3 chemokines on CLAD risk

We hypothesized that episodes of OP with high BALF CXCR3 ligand concentrations represent greater Type I immune response involvement and would be associated with increased CLAD risk. To explore this association, a time-dependent cumulative variable for OP was created using quartiles of CXCL9, CXCL10 and CXCL11 concentrations. Episodes of OP with higher CXCR3 ligand concentrations were associated with a significant increase in CLAD risk. In the univariable model, the HR for CLAD for an episode of OP with CXCL9 concentration greater than the 25^th^ percentile (203.1 pg/ml) was 1.6 (95% CI 1.1–2.4, Table 3). Using the 50^th^ (1139.9 pg/ml) and 75^th^ (4493.1 pg/ml) percentile cutoffs for CXCL9, the HR for CLAD increased to 2.2 (95% CI 1.4–3.4) and 2.3 (95% CI 1.4–3.9), respectively. In the multivariable model adjusted for other injury patterns, OP with CXCL9 concentrations greater than the 25^th^, 50^th^, and 75^th^ percentiles had HRs of 1.4 (95% CI 0.92–2.1), 1.9 (95% CI 1.2–2.9) and 1.8 (95% CI 1.1–3.2), respectively.

Similarly, episodes of OP with higher BALF CXCL10 and CXCL11 concentrations were associated with higher HRs for CLAD. In multivariable models, OP with CXCL10 greater than the 25^th^ (79.8 pg/ml), 50^th^ (286.7 pg/ml) and 75^th^ (810.2 pg/ml) percentiles had HRs for CLAD of 1.4 (95% CI 0.95–2.1), 1.8 (95% CI 1.2–2.8) and 1.8 (95% CI 1.1–3.2), respectively (Table 4). Similarly, OP with CXCL11 concentrations greater than the 25^th^ (13.9 pg/ml), 50^th^ (67.5 pg/ml) and 75^th^ (106.0 pg/ml) percentile cutoffs had adjusted HRs for CLAD of 1.6 (95% CI 1.1–2.4), 1.8 (95% CI 1.2–2.8) and 1.7 (95% CI 0.93–3.1), respectively (Table 5).

**Table 4:** Cox proportional hazards model for CLAD. Using BAL CXCL10 concentrations cutoffs.

	Univariable		Multivariable ƚ	
	HR (95% CI)	p-value	HR (95% CI)	p-value
**OP**	1.55 (1.13–2.12)	0.007	1.34 (0.97–1.86)	0.077
**OP with CXCL10 > 25th%**	1.68 (1.14–2.48)	0.009	1.42 (0.95–2.12)	0.090
**OP with CXCL10 > 50th%**	2.09 (1.36–3.20)	0.001	1.82 (1.18–2.82)	0.007
**OP with CXCL10 > 75th%**	2.29 (1.35–3.89)	0.002	1.83 (1.06–3.16)	0.031

Definition of abbreviations: CLAD = chronic lung allograft dysfunction; HR = hazards ratio; CI = confidence interval; OP = organizing pneumonia; % = percentile.

^ƚ^ Multivariable model adjusted for diffuse alveolar damage, acute rejection, and lymphocytic bronchiolitis.

**Table 5:** Cox proportional hazards model for CLAD. Using BAL CXCL11 concentrations cutoffs.

	Univariable		Multivariable ƚ	
	HR (95% CI)	p-value	HR (95% CI)	p-value
**OP**	1.55 (1.13–2.12)	0.007	1.34 (0.97–1.86)	0.077
**OP with CXCL11 > 25th%**	1.85 (1.26–2.73)	0.002	1.60 (1.08–2.39)	0.020
**OP with CXCL11 > 50th%**	2.09 (1.37–3.21)	0.001	1.80 (1.16–2.79)	0.009
**OP with CXCL11 > 75th%**	2.13 (1.19–3.84)	0.011	1.70 (0.93–3.10)	0.085

Definition of abbreviations: CLAD = chronic lung allograft dysfunction; HR = hazards ratio; CI = confidence interval; OP = organizing pneumonia; % = percentile.

^ƚ^ Multivariable model adjusted for diffuse alveolar damage, acute rejection, and lymphocytic bronchiolitis.

PC analysis was used to assess the three chemokines in aggregate and confirmed the higher CLAD risk associated with elevated BALF CXCR3 chemokine concentrations. In the univariable models, the HRs increased from 1.8 (95% CI 1.2–2.7), 2.2 (95% CI 1.4–3.2), and 2.6 (95% CI 1.7–4.0) for OP using the 25^th^, 50^th^, and 75^th^ percentile cutoffs for the PC, respectively (Table 6). Similarly, in the multivariable models, the HRs increased from 1.5 (95% CI 1.0–2.3), 1.9 (95% CI 1.2–2.8), and 2.2 (95% CI 1.4–3.4) for OP using the quartile cutoffs.

**Table 6:** Cox proportional hazards model for CLAD. Using BAL CXCR3 ligand concentration principal component cutoffs.

	Univariable		Multivariable ƚ	
	HR (95% CI)	p-value	HR (95% CI)	p-value
**OP**	1.55 (1.13–2.12)	0.007	1.34 (0.97–1.86)	0.077
**OP with PC > 25th%**	1.83 (1.25–2.69)	0.002	1.55 (1.04–2.31)	0.032
**OP with PC > 50th%**	2.15 (1.44–3.21)	0.001	1.86 (1.23–2.80)	0.003
**OP with PC > 75th%**	2.56 (1.65–3.95)	0.001	2.16 (1.37–3.39)	0.001

Definition of abbreviations: CLAD = chronic lung allograft dysfunction; HR = hazards ratio; CI = confidience interval; OP = organizing pneumonia; PC = First principal component; % = percentile.

^ƚ^ Multivariable model adjusted for diffuse alveolar damage, acute rejection, and lymphocytic bronchiolitis.

**Fig 3:**
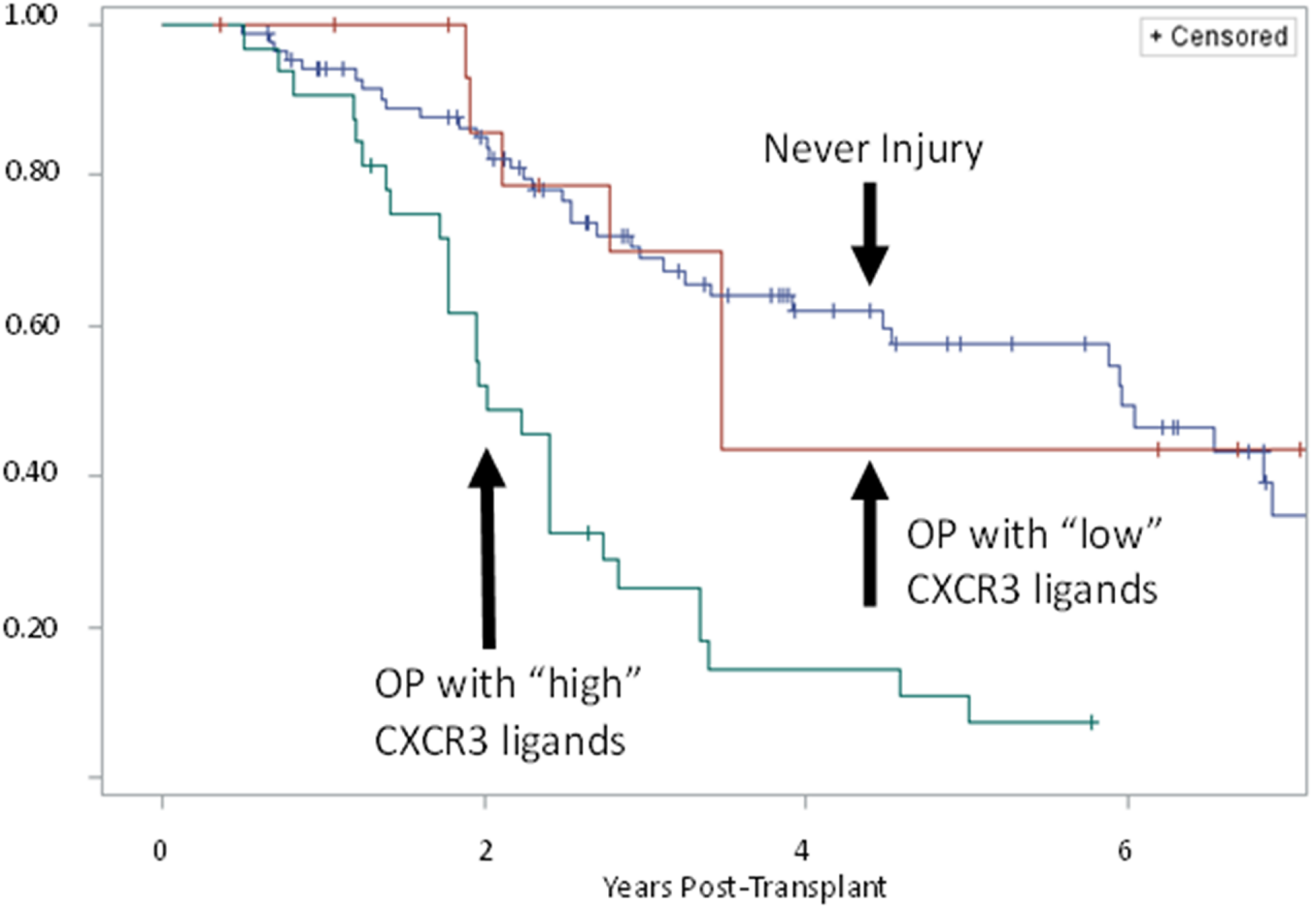
Kaplan-Meier plot for freedom from chronic lung allograft dysfunction (CLAD) by lung transplant recipients who: 1) Never had any allograft injury, 2) Developed OP with “low” BAL CXCR3 ligand concentrations (“low” ≤ median first principal component (PC) of three CXCR3 ligands), 3) Developed OP with “high” BAL CXCR3 ligand concentrations (“high” > median PC of three ligands).

A Kaplan-Meier curve for freedom from CLAD was constructed stratified by whether a recipient had: 1) an episode of OP with BALF CXCR3 ligand PC level greater than the median, 2) an episode of OP with BALF CXCR3 ligand PC level lower than (or equal to) the median, or 3) no history of histopathologic injury (OP, DAD, AR or LB). The 5-year incidence of CLAD was 89% among recipients who had an episode of OP with “high” BALF CXCR3 ligand levels vs. 56% among recipient who had an episode of OP with “low” CXCR3 ligand levels vs. 43% among recipients with no history of histopathologic injury (Fig 3). There was a significant difference between recipients who had an episode of OP with high BALF CXCR3 ligands compared with those who had OP but without high CXCR3 ligands (p = 0.0021). Recipients who had OP without high CXCR3 ligands were not significantly different from those without allograft injury (p = 0.9385).

### Impact of high BALF CXCR3 chemokines on RAS / BOS risk

Given its alveolar and parenchymal location, we hypothesized that OP would be associated with RAS, the restrictive phenotype of CLAD. In a subset analysis among double LTRs with chest CTs within 3 months of CLAD diagnosis, we explored the association between OP, BALF CXCR3 ligands and the CLAD phenotypes: RAS and BOS. Of the 202 double LTRs with sufficient spirometric and radiographic data, 106 (53%) developed CLAD. Fifty-one (48%) of these recipients met RAS criteria, while the remaining 55 (42%) were categorized BOS. The univariable models for time to RAS showed a similar pattern as time to CLAD, but with higher HRs for all BALF chemokine level cutoffs. In the univariable model, an episode of OP without regard for BALF chemokines was associated with RAS development: HR 2.4 (95% CI 1.4–4.3, Table 7). The HRs were 2.4 (95% CI 1.2–4.8), 2.8 (95% CI 1.4–5.6) and 4.1 (95% CI 2.0–8.4) using the 25^th^, 50^th^ and 75^th^ percentile cutoffs for the PC. For the multivariable model adjusted for the other injury patterns, the HRs was 1.9 (95% CI 1.1–3.5) for an episode of OP without regard to BALF chemokines. The HRs were 1.9 (95% CI 0.9–3.8), 2.2 (95% CI 1.0–4.5) and 3.0 (95% CI 1.4–6.6) using the 25^th^, 50^th^ and 75^th^ percentile cutoffs for the PC in the multivariable model, respectively.

**Table 7:** Cox proportional hazards model for RAS. Using BAL CXCR3 ligand concentration principal component cutoffs.

	Univariable		Multivariable ƚ	
	HR (95% CI)	p-value	HR (95% CI)	p-value
**OP**	2.41 (1.37–4.25)	0.002	1.91 (1.05–3.45)	0.033
**OP with PC > 25th%**	2.38 (1.19–4.75)	0.014	1.86 (0.90–3.83)	0.092
**OP with PC > 50th%**	2.82 (1.41–5.64)	0.003	2.16 (1.05–4.46)	0.037
**OP with PC > 75th%**	4.06 (1.97–8.37)	0.001	3.03 (1.39–6.60)	0.005

Definition of abbreviations: CLAD = chronic lung allograft dysfunction; HR = hazards ratio; CI = confidience interval; OP = organizing pneumonia; PC = First principal component; % = percentile.

^ƚ^ Multivariable model adjusted for diffuse alveolar damage, acute rejection, and lymphocytic bronchiolitis. Double lung transplant recipients only.

The association between OP, BALF CXCR3 ligands and BOS development was weaker than that for RAS development. In the univariable model, an episode of OP without regard for BALF chemokines was not associated with BOS development: HR 0.8 (95% CI 0.4–1.6, Table 8). There was an increase in HRs with higher BALF CXCR3 chemokines: 1.7 (95% CI 0.8–3.6), 2.0 (95% CI 0.9–4.3), and 2.5 (95% CI 1.1–5.9) for the 25^th^, 50^th^ and 75^th^ percentile cutoffs for the PC, respectively. However, these variables did not reach statistical significance as predictors of BOS in the multivariable models.

**Table 8:** Cox proportional hazards model for BOS. Using BAL CXCR3 ligand concentration principal component cutoffs.

	Univariable		Multivariable ƚ	
	HR (95% CI)	p-value	HR (95% CI)	p-value
**OP**	0.76 (0.37–1.56)	0.449	0.68 (0.32–1.44)	0.317
**OP with PC > 25th%**	1.69 (0.80–3.59)	0.171	1.65 (0.75–3.60)	0.211
**OP with PC > 50th%**	2.00 (0.94–4.25)	0.071	1.95 (0.89–4.25)	0.096
**OP with PC > 75th%**	2.52 (1.07–5.92)	0.034	2.41 (0.98–5.91)	0.055

Definition of abbreviations: CLAD = chronic lung allograft dysfunction; HR = hazards ratio; CI = confidience interval; OP = organizing pneumonia; PC = First principal component; % = percentile.

^ƚ^ Multivariable model adjusted for diffuse alveolar damage, acute rejection, and lymphocytic bronchiolitis. Double lung transplant recipients only.

## Discussion

The identification and study of key risk factors of CLAD pathogenesis is a critical step towards improving outcomes after lung transplantation. Prior studies have established AR, LB and DAD as strong risk factors for CLAD development [9,13,14,23,24]. However, there is a paucity of studies evaluating the association between OP and CLAD. In this study, we evaluated the association between OP and CLAD using multivariable analysis adjusting for other allograft injury patterns (DAD, AR, LB) as well as the prognostic significance of BALF CXCR3 chemokine concentrations. We evaluated 690 BALF samples from 324 recipients and found marked elevations in all three CXCR3 ligands during OP compared with “healthy” samples. In multivariable models adjusted for other injury patterns, OP did not significantly increase the risk of CLAD when BAL CXCR3 chemokine concentrations *were not taken into account*. However, OP with elevated CXCR3 ligands markedly increased CLAD risk in a dose-response manner. An episode of OP with CXCR3 concentrations greater than the 25^th^, 50^th^ and 75^th^ percentiles had HRs for CLAD of 1.5 (95% CI 1.0–2.3), 1.9 (95% CI 1.2–2.8) and 2.2 (95% CI 1.4–3.4), respectively. This study identifies OP, a relatively uncommon histopathologic finding after lung transplantation as a major risk factor for CLAD development when considered in the context of increased allograft expression of interferon-γ inducible ELR- CXC chemokines. Furthermore, it demonstrates for the first time, the prognostic importance of BALF CXCR3 ligand concentrations during OP on subsequent CLAD risk.

There have only been a few prior studies evaluating the association between OP and CLAD. Girgis et al evaluated 74 LTRs and found no association between 22 episodes of OP and CLAD in univariable or multivariable analysis.[19] A larger study involving 230 LTRs similarly did not find OP to be an independent predictor of CLAD.[22] Of note, OP is a histologically distinct entity from acute fibrinoid organizing pneumonia (AFOP) which was recently described and characterized by alveolar deposition of fibrillary fibrin without interstitial mononuclear cell infiltration.[33] We previously evaluated 1894 TBBXs from 441 LTRs and found that although OP predicts CLAD development in univariable analysis, it loses significance after multivariable adjustment for the other injury patterns: DAD, AR and LB.[24]

We hypothesized that a Type I immune response involving CXCR3/ligand biology would be augmented during episodes of OP, compared with “healthy” biopsies. Histopathologically, OP is characterized by interstitial and alveolar mononuclear cell infiltration with excessive proliferation of fibroblasts and granulation tissue predominantly within the alveoli. CXCR3 ligands have been shown to act as potent chemoattractants for mononuclear cells [34,35] and we have previously demonstrated the importance of the CXCR3/ligand biological axis in the pathogenesis of AR and CLAD in both animal and clinical studies.[25,26] We furthermore hypothesized that episodes of OP with higher BALF CXCR3 ligand concentrations represent greater Type I immune response involvement, and would therefore have a higher risk of subsequent CLAD development. PC analysis was used to assess the three chemokines in aggregate and demonstrated a marked increase in CLAD risk with increasing CXCR3 chemokine concentrations during OP. Importantly, in both univariable and multivariable models, the risk of CLAD increased with higher CXCR3 ligand concentrations in a dose-response relationship.

Kaplan Meier analysis showed a significant difference in time to CLAD between recipients who had an episode of OP with “high” CXCR3 ligand concentrations (>50^th^ percentile) and those who had OP with “low” CXCR3 ligands. However, there was no statistically significant difference between recipients who had OP with “low” CXCR3 ligands (≤50^th^ percentile) and recipients who never developed any allograft injury. These findings support the role of CXCR3/ligand biologic axis in the continuum of OP to CLAD, as well as the potential use of BALF CXCR3 ligands as a prognostic biomarker after lung transplantation.

Immunohistochemistry studies demonstrate CXCL9 and CXCL10 expression predominately by reactive type 2 pneumocytes, CXCL11 by endothelial cells, and CXCR3 by infiltrating parenchymal mononuclear cells including macrophages. Collectively, this supports a coordinated cascade by chemokines. CXCL11 recruits CXCR3 expressing mononuclear cells and macrophages from the allograft vasculature. Once in the lung, these cells move down a CXCL9 and CXCL10 chemokine gradient to sites of allograft injury.

The major limitation of this study is the potential for confounding given its retrospective single center design. For example, patients with clinical deterioration may have received more frequent biopsies leading to a higher incidence of OP as well as higher BALF CXCR3 ligand concentrations. Our study design accounted only for allograft injuries that were captured by TBBX. Undoubtedly, there were episodes that were missed due to the infrequency of TBBX sampling and poor sensitivity for detecting more subtle injury patterns. Importantly, maintenance immunosuppression, azithromycin use, and treatments received for allograft injury were also not taken into account. At our institution, patients routinely received augmented immunosuppression for AR, but for DAD, OP and LB, it is left to the discretion of the lung transplant physician. Lastly, this study included both surveillance and clinically indicated bronchoscopies. Sample size limitation did not allow for stratification by clinical indication.

This study improves our understanding of CLAD pathogenesis. The identification of key events which increase CLAD risk represents unique opportunities to better understand the immunologic processes responsible for CLAD development. We identify another strong histopathologic risk factor for CLAD development and provide support for our hypothesis regarding a potential mechanism of CLAD pathogenesis: the role of aberrant CXCR3/ligand biology in the propagation of allograft injury. The CXCR3 ligands likely perpetuate allograft injury by recruiting injurious mononuclear cells and macrophages into the allograft causing further cell damage, Type I immune response with augmentation of CXCR3 ligand release, additional mononuclear cell / macrophage recruitment leading to eventual fibroproliferation. To our knowledge, this is the first study to evaluate the association between OP and CLAD, taking into account the prognostic significance of BALF CXCR3 ligand concentrations. This study evaluated 690 BALF samples from 324 recipients and is one of the largest studies to evaluate chemokine expression patterns during allograft injury.

In summary, we evaluated the association between OP and CLAD taking into account all four injury patterns as well as the prognostic significance of BALF CXCR3 ligands. We found marked elevations of all three CXCR3 ligands during OP compared with “healthy” samples, and demonstrated that CXCR3 ligand elevation during OP significantly increases the risk of subsequent CLAD development, more specifically the restrictive phenotype RAS, in a dose-response manner. These findings demonstrate the prognostic importance of BALF CXCR3 ligands and identifies OP in the context of elevated BALF CXCR3 ligands as a major predictor of CLAD development. Future studies should evaluate the pharmacologic disruption of this pathway as a strategy to reduce the incidence of CLAD.

## Supporting information

S1 Table Wideall3.sas7bdat. Dataset of pathologic injuries, chemokines and outcomes in wide format.
(SAS7BDAT)

S2 Table Pathcyto3.sas7bdat. Dataset of pathologic injuries and chemokines in long format.
(SAS7BDAT)
